# Expression and Purification of a PEDV-Neutralizing Antibody and Its Functional Verification

**DOI:** 10.3390/v13030472

**Published:** 2021-03-12

**Authors:** Wenshu Shi, Haiyang Hao, Mengran Li, Jianqin Niu, Yaning Hu, Xingbo Zhao, Qiuyan Li

**Affiliations:** 1 College of Animal Science and Technology, China Agricultural University, Beijing 100193, China; shiwenshu1992@163.com (W.S.); huyaning17@163.com (Y.H.); 2 College of Biological Sciences, China Agricultural University, Beijing 100193, China; haohaiyanghhy@cau.edu.cn (H.H.); lmr_hbu@163.com (M.L.); 18332568575@163.com (J.N.)

**Keywords:** PEDV, neutralizing antibody, eukaryotic expression vector, oral administration

## Abstract

Porcine epidemic diarrhea virus (PEDV) is a highly infectious and pathogenic virus causing high morbidity and mortality, especially in newborn piglets. There remain problems with contemporary PEDV vaccines, in part because of the rapid variation of PEDV, poor conferred immunity, and numerous side effects. The ability to produce PEDV-neutralizing antibodies suggests that we may be able to increase the success rate of PEDV prevention in piglets using these antibodies. In this study, we produced an anti-PEDV S protein monoclonal antibody (anti-PEDV mAb-2) that neutralized PEDV-CV777 (a G1 strain), PEDV-SDSX16 and PEDV-Aj1102 (two G2 strains). In vivo challenge experiments demonstrated that anti-PEDV mAb-2 inhibited the PEDV infection in piglets. We also produced three HEK293 cell lines that expressed anti-PEDV mAb-2. Overall, our study showed that anti-PEDV mAb-2 produced from hybridoma supernatants effectively inhibited PEDV infection in piglets, and the recombinant HEK293 cell lines expressed anti-PEDV mAb-2 genes.

## 1. Introduction

Porcine epidemic diarrhea (PED) is a highly infectious diarrhoeal disease in pigs caused by the porcine epidemic diarrhea virus (PEDV). It is characterized by acute watery diarrhea, vomiting and dehydration with high mortality, especially in newborn and weaned piglets [1]. The first outbreak of PED was recorded in Europe in the early 1970s and has since spread to Asia and North America [2]. Since the 1990s, large-scale PEDV outbreaks have been reported in several countries in Asia [3]. From 2017 and 2019, PEDV was still the primary pathogen causing porcine diarrhea in China [4]. The virus has caused extremely high mortality and serious economic damage to the pig industry.

PEDV is an enveloped, positive-sense and single-stranded RNA virus that belongs to the order *Nidovirales*, suborder *Cornidovirineae*, family *Coronaviridae*, subfamily *Orthocoronavirinae* and genus *Alphacoronavirus* [5]. Its genome is about 28 kb, with a 5′ cap and a 3′ polyadenylated tail [1]. PEDV has seven open reading frames (ORFs) encoding for three nonstructural proteins responsible for viral genome replication and transcription, and four structural proteins: spike protein (S), envelope protein (E), membrane glycoprotein (M) and nucleocapsid protein (N). The S protein is a major type 1 membrane glycoprotein on the viral surface, 1383–1386 amino acids in length. Among the structural proteins, the S protein plays a central role in the infection of host cells because of its interaction with cell membrane receptors, and its ability to induce neutralizing antibodies in host animals [6]. According to the phylogenetic analysis of the full-length of S gene, PEDV are divided into two subtypes of G1 and G2 [7], G1 is mainly represented by CV777 strains [8]. The mutant strains SDSX16/JX/Aj1102 in most Asian countries belong to G2 subtypes [9,10,11].

During infection the S protein is cleaved into the S1 (aa 1–789) and S2 domains (aa 790–1383); the S1 domain contains major neutralizing epitopes [12], and is a suitable region for determining genetic correlations between different isolates and conducting differential PEDV diagnostic tests. Taking into account these molecular and biological properties, the S1 domain is a suitable target for developing effective PEDV vaccines [13].

Inactivated and attenuated vaccines are widely used in most countries around the world, but repeated outbreaks of PEDV on large farms and the emergence of highly pathogenic strains, indicate that the effectiveness of vaccination is not complete. Inactivated (or subunit vaccines) elicit mainly IgG antibodies in serum but do not induce mucosal immunity, resulting in little maternal antibody available in colostrum [14]. Additionally, as PEDV mainly infects and replicates in the villus epithelium of the small intestine, these vaccines do not result in an ideal therapeutic effect [15]. Passive lactogenic immunity remains the principal way of protecting piglets from PEDV [16], but because of vaccination deficiencies, the serious pathogenicity of virus, and the incomplete development of the immune system of suckling piglets, they still suffer very high mortality rates from PEDV [4,17]. These issues have prompted many scholars to investigate methods for improving the immune effect from oral immunization [18,19].

To develop an effective alternative to current PEDV vaccines, we prepared a monoclonal antibody with PEDV neutralizing activity. Two eukaryotic expression vectors were constructed, one containing the Fc and light chain sequences, and the other containing the Fc and the heavy chain sequences of the monoclonal antibody. We then produced three HEK293 cell lines that expressed anti-PEDV mAb-2 genes. In vivo PEDV challenge experiments showed that oral administration of the antibody inhibited PEDV infection in newborn piglets.

## 2. Materials and Methods

### 2.1. Ethics Statement of Animal Usage

All animal studies and experimental procedures were approved by the Committee on the Ethics of Animal Experiments of China Agricultural University (Permit Number: AW72101202-1-2). The experimental animals were housed in the Laboratory Animal Centre under environmental parameters of 12 h alternating light/dark, 20–26 °C ambient temperature, 40–70%, humidity, HEPA-filtered air was provided, and air cleanliness was 7.

### 2.2. Cells, Virus, and Protein for Immunity

Vero cells and HEK293 cells were from the National Animal Gene Research Center of China Agricultural University. Cells were maintained in Dulbecco’s modified Eagle medium (DMEM) supplemented with 10% heat-inactivated fetal bovine serum (FBS) and antibiotics (100 U/mL of penicillin and 8 μg/mL of streptomycin) (Gibco, CA, USA) in a humidified 5% CO_2_ incubator at 37 °C. Maintenance medium without FBS and supplemented with trypsin (7.5 μg/mL) (Gibco, CA, USA) was used for the preparation of virus cultures and virus-neutralizing assays (VN). PEDV S protein was expressed in BL21 strain at HuaDa Protein Research and Development Center (Beijing, China).

### 2.3. Generation of PEDV Virus Stocks

Three PEDV strains from different genogroups were used in this study: the G1 strain, PEDV-CV777 (GenBank Accession No. KU664503), PEDV-SDSX16 (G2 strain) isolated from a naturally infected piglet, PEDV-Aj1102 (G2 strain) originated from a commercial vaccine of Ke Qian (China). PEDV strains were grown on monolayers of Vero cells grown to 70% confluency in T25 flasks according to the method by Hofmann M with some modifications [20]. Briefly, cells were washed with PBS, then the virus, at a multiplicity of infection (MOI) of 0.1, was added to each flask and incubated at 37 °C in 5% CO_2_ incubator. After 2 h post-infection (hpi) the virus inoculum was removed and the maintenance medium was added back. When cytopathic effect (CPE) was evident (approximately 72 hpi), virus cultures were harvested with three freeze-thaw cycles then centrifuged for 10 min at 1000 rpm to remove cell debris. Virus titers were determined by endpoint dilution in Vero cells and expressed as 50% tissue culture infective dose (TCID_50_). Virus stocks were stored at −80 °C until needed.

### 2.4. Preparation of PEDV S-Specific mAbs with Neutralizing Activity

Four 6-week-old female Balb/c mice were immunized by subcutaneous injection with 60 μg PEDV S protein. Each mouse also received three subcutaneous immunizations with 30 μg polypeptide at two-week intervals. Seven days after the third injection, serum antibody titers, from orbital blood samples, were determined by enzyme-linked immunosorbent assay (ELISA) using PEDV S as antigen. The mouse with the highest antibody titer was boosted with an intraperitoneal injection of 50 μg polypeptide. Three days after injection, the mouse was sacrificed, the spleen was collected, and erythrocyte- and monocyte-depleted spleen cell populations were prepared. After gentle washing with brief centrifugation, splenocytes were fused with SP2/0 at a cell ratio of approximately 10: 1 using polyethylene glycol 2000 (Sigma-Aldrich, St. Louis. MO, USA). Hybridomas were seeded onto cell culture plates in semisolid medium supplemented with Hypoxanthine, Aminopterin, and Thymidine (HAT) medium (Sigma-Aldrich, St. Louis. MO, USA), 20% FBS, 100 U/mL of penicillin and 100 mg/mL of streptomycin (Gibco, Waltham, CA, USA), and incubated at 37 °C in a humidified incubator with 5% CO_2_ for about 10 days, as described previously with some modifications [21]. The hybridoma culture supernatants were screened for the production of PEDV S-specific antibodies using ELISA, and the specific antibody-producing hybridoma cultures were cloned by sorting into 96-well plates and tested for reactivity by PEDV neutralizing assay.

### 2.5. Preparation of Ascitic Fluid in Perioneum and Antibody Purification

The hybridoma clone producing antibody with the highest neutralizing activity was expanded and transplanted into the mice for ascites production. Cell density was adjusted to 6 × 10^6^/mL, and 0.2 mL were injected intraperitoneally into each Balb/c mouse that had been primed with liquid paraffin oil. Ascites were collected 7–10 days after injection and its neutralizing activity was tested [22]. Ascites were purified using saturated ammonium sulfate according to the manufacturer’s protocol (Sangon Biotech, Shanghai, China).

### 2.6. Enzyme-Linked Immunosorbent Assay (ELISA)

The purified soluble PEDV S protein was diluted in buffer (50 mM Na_2_CO_3_, 50 mM NaHCO_3_, pH 9.6) to a final concentration of 2 μg/mL, and 100 μL was used to coat each well of microtiter plates (Corning, NY, USA) overnight at 4 °C. Plates were then washed three times with PBST (PBS + 0.05% Tween) and blocked with 5% non-fat milk in PBST for 3 h at 37 °C. Plates were washed three times, then 100 µL of hybridoma supernatant or diluted mouse serum was aliquoted to each well and incubated for 1 h at 37 °C. After three washing steps, 100 µL of a 1: 20,000 dilution of horseradish peroxidase (HRP)-conjugated rabbit anti-mouse IgG (Dako, Danmark) was aliquoted to each well and incubated for 1 h at 37 °C. After three washes with PBST, 1 × TMB (Solabio, Shanghai, China) substrate was aliquoted per well and incubated for 15 min at 37 °C. The reaction was stopped by the addition of 2 M H_2_SO_4_ [15]. Absorbance at 450 nm was measured using a CMax Plus ELISA reader (Molecular Devices, Beijing, China).

### 2.7. Neutralization Test

The neutralizing antibody titers of PEDV in hybridoma cell supernatants were determined according to the method by Chunhua Li et al. [12] with some modifications. Briefly, 2 × 10^5^ cells were inoculated into wells of six-well cell culture plates and incubated for 72 h. Supernatants were collected, filtered through a 0.22 μm membrane, then serially diluted 2-fold (the antibody dilution range was from 1:2 to 1:128, and the mouse ascites dilution range was from 1:2 to 1:2048). 200 μL of each dilution was mixed with an equal volume of 200 TCID_50_ PEDV strain and incubated for 1 h at 37 °C to allow virus-antibody complexes to form. Monolayers of Vero cells in 96-well plates were washed 3 times with phosphate buffer saline (PBS) then inoculated with the virus-antibody complexes and incubated for 1 h at 37 °C. A positive control (virus only, no mAb), a negative control (virus, non-related cell supernatant) and a mock group (no virus, no mAb) were included on each plate. Cells were washed again to remove unabsorbed virus then incubated in a maintenance medium at 37 °C in 5% CO_2_. CPE was observed after 5–7 days, and the neutralizing concentration was defined as the lowest concentration of antibodies that prevented the occurrence of CPE. Three PEDV strains (PEDV-CV777, PEDV-SDSX16 and PEDV-Aj1102) were used as antigens in the neutralization test.

### 2.8. Construction of Eukaryotic Expression Vectors Containing Anti-PEDV mAb-2 Genes

**Figure 1:**
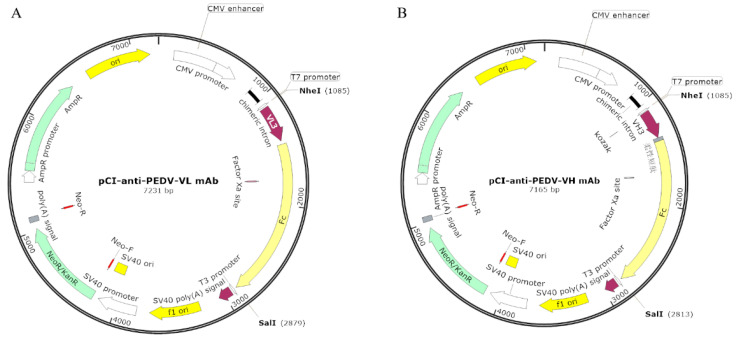
The vector map of eukaryotic expression vectors. (**A**) pCI-anti-porcine epidemic diarrhea virus (PEDV)-VL mAb. (**B**) pCI-anti-PEDV-VH mAb.

The pCI-anti-PEDV-VL mAb and pCI-anti-PEDV-VH mAb vectors are modifications of pCI-Neo-hTERT from State Key Laboratory for Agrobiotechnology, China Agricultural University. Briefly, the heavy and light chains of mAb-2 were amplified and fused to Fc fragments by PCR. Each amplicon contained a 5′ *Nhe* I site and a 3′ *Sal* I site. Each amplicon was ligated into *Nhe* I/*Sal* I linearized pCI-Neo-hTERT. The recombinant plasmids pCI-anti-PEDV-VL mAb and pCI-anti-PEDV-VH mAb were extracted using a Plasmid Maxi Kit (Tiangen Biotech, Beijing, China). Figure 1A,B are the vector maps.

### 2.9. Cell Transfection and Selection

According to the instructions provided by the Amaxa Basic Nucleofector Kit (Lonza, VPI-1002), HEK293 cells were transfected by electroporation. Three micrograms of pCI-anti-PEDV-VL and pCI-anti-PEDV-VH were added to 200 μL electroporation medium and mixed with approximately 1 × 10^4^ cells and transferred into a Lonza cuvette for electroporation. After electroporation 1 mL of DMEM was added to each reaction mix then aliquoted into multiple 10 cm plates with 900 μg/mL G418 and cultured for 7 days. The G418 resistant clones with good morphology were expanded for genome extraction and cryopreservation.

### 2.10. Oral Antibody Test in Piglets

**Figure 2:**
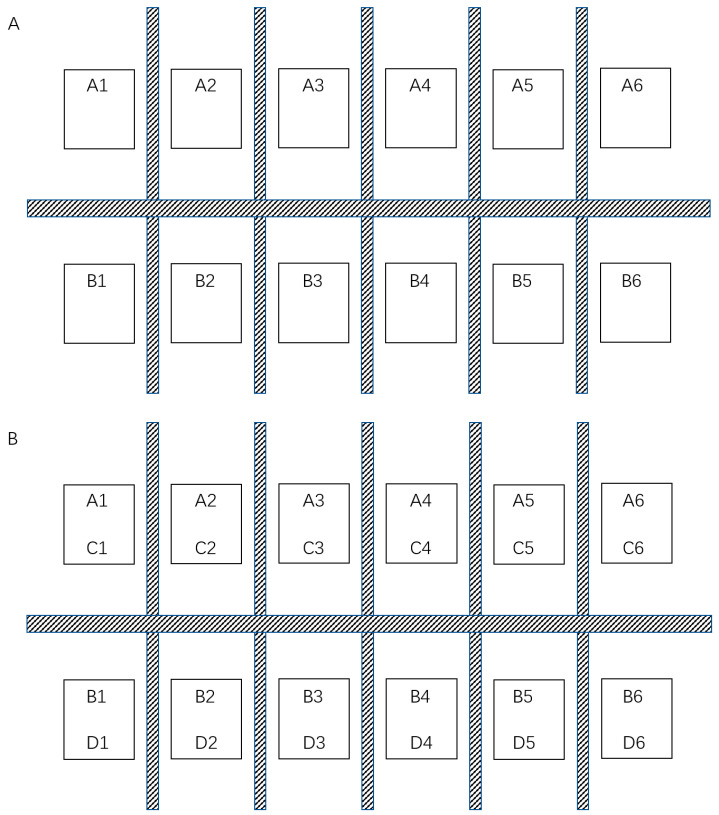
The design for in-vivo test distribution map. (**A**) The distribution of piglets in group A and group B. (**B**) The distribution of piglets in group A, group B, group C and group D. The combination of letters and numbers in the figure represents the piglet number in each group.

To test whether the anti-PEDV mAb-2 can protect piglets from the viral challenge in vivo, 12 newborn Large White piglets, negative for PEDV, TGEV, PCV and PRV antigens (Oligonucleotides used for PCR were in Table S1) and PEDV antibodies, were divided into 2 treatment groups randomly. Each piglet in group A received 3 mL of anti-PEDV mAb-2 as a single does, and each piglet in group B received 3 mL DMEM as a single does; all doses were received orally. These piglets were housed in separate pens (The piglet distribution was shown in Figure 2A). The additional 12 Large White piglets did not oral anti-PEDV mAb-2, whose mother suffered PED, were then cohoused with group A and B piglets respectively (6 piglets per group, the 6 piglets cohoused with group A named group C, the 6 piglets cohoused with group B named group D). The piglet distribution was illustrated in Figure 2B. All the tested pigs were male, before weaning. They were observed daily at 3:00 pm for 30 min. For each piglet, the degree of diarrhea was assessed as described by Lijuan Yuan [23] with some modifications: 1, normal; 2, pasty; 3, semiliquid; and 4, liquid (Table 1). To avoid cross-infection among individuals in group A and group B, strict attention was paid to the hygiene management of the piglet housing and care staff.

### 2.11. Isolation of RNA and cDNA, and RT-PCR

Total virus RNA (vRNA) from infected-cell supernatants was isolated using TRIzol reagent (Invitrogen, Carlsbad, CA, USA) according to the manufacturer’s instructions. Complementary DNA (cDNA) was produced by reverse transcription, using Maxima H Minus First Strand cDNA Synthesis Kit (ThermoFisher, Waltham, MA, USA) according to the manufacturer’s instructions. KOD One Mix polymerase (Toyobo, Shanghai, China) was used for RT-PCR.

### 2.12. Statistical Analysis

Statistical comparisons were analyzed using GraphPad Prism (Version 7.00) software. The differences between the treatment group and the control group in IgG and neutralizing antibody were measured by ANOVA or Mann–Whitney accordingly. Differences were considered significant if the *p*-value was <0.05. The *p*-values are indicated as follows: * *p* < 0.05; ** *p* < 0.01; *** *p* < 0.001.

## 3. Results

### 3.1. Screening of PEDV S Protein-Positive Hybridomas

**Figure 3:**
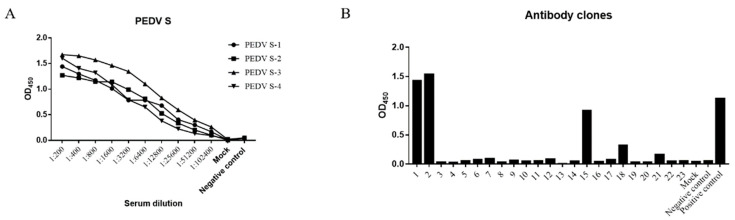
Measurement of anti-PEDV mAb-2. (**A**) Enzyme-linked immunosorbent assay (ELISA) measurement of serum IgG titers of the four immunized mice. (**B**) ELISA measurement of IgG in the supernatants of 23 hybridoma clones, the serum of mouse-3 was used as a positive control. The numbers of positive hybridoma cells were 1, 2, 15, 18 and 21. Their ODs450 were 1.462, 1.532, 0.914, 0.326 and 0.162 respectively.

Figure 3A shows the serum IgG antibody titers in the mice immunized with PEDV S protein, as determined by ELISA. At this point, mouse-3 had produced anti-PEDV antibodies with the highest binding activity; this mouse was boosted and sacrificed 4 days later for the production of hybridomas. Figure 3B shows the ELISA results of 23 hybridoma clones screened by ELISA. The genotypes of the antibody heavy and light chains produced by clones 1, 2, 15, 18, and 21 are shown in Table S2.

### 3.2. Neutralization Test

**Figure 4:**
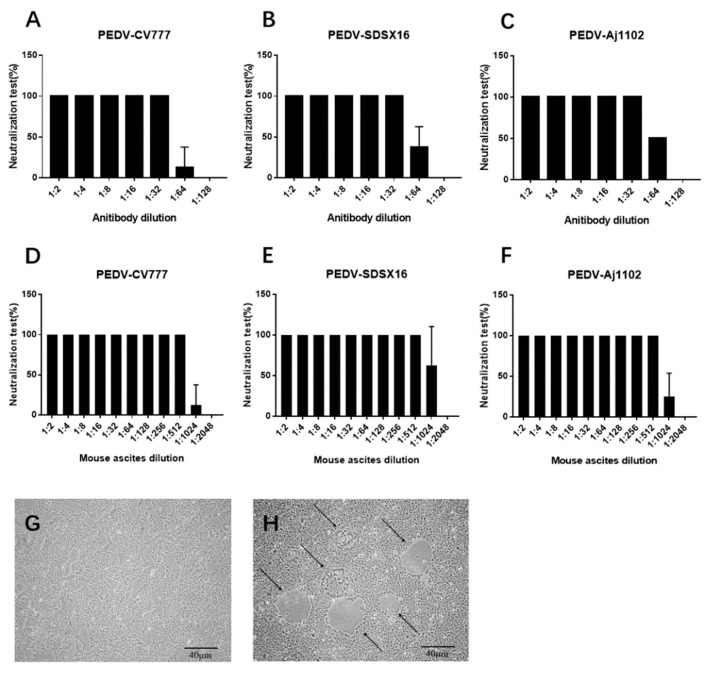
The neutralization of PEDV by anti-PEDV mAb-2 from hybridoma supernatant and mouse ascites. (**A**–**C**) Neutralization of PEDV-CV777, PEDV-SDSX16 and PEDV-Aj1102 by anti-PEDV mAb-2 hybridoma supernatant. (**D**–**F**) Neutralization of PEDV-CV777, PEDV-SDSX16 and PEDV-Aj1102 by anti-PEDV mAb-2 mouse ascites. (**G**) Morphology of Vero cells after incubation for 72 h with anti-PEDV mAb-2 and PEDV. (**H**) Morphology of Vero cells after incubation for 72 h with PEDV only. The black arrow indicates the presence of syncytial bodies. Each column represents the average of triplicates, and each error bar indicates the standard deviations.

Supernatants from hybridomas 1, 2, 15, 18, and 21 were tested for neutralization activity against PEDV infection, only anti-PEDV mAb-2 demonstrated a protective affect against infection in Vero cells. Figure 4 A–C shows the neutralizing activity of anti-PEDV mAb-2 from supernatant against PEDV-CV777, PEDV-SDSX16 and PEDV-Aj1102 respectively. Note that the dilution for 100% neutralization against all strains was 1: 32. Figure 4 D–F shows the neutralizing activity of anti-PEDV mAb-2, purified from mouse ascites, against each of the PEDV strains. Figure 4G shows Vero cells incubated with anti-PEDV mAb-2 and PEDV, the morphology of cells is normal. Figure 4H shows Vero cells incubated with PEDV only, here the cells are shrunken, forming syncytial bodies, and detached.

### 3.3. Highly Efficient Construction Of Recombinant Expression Vector

**Figure 5:**
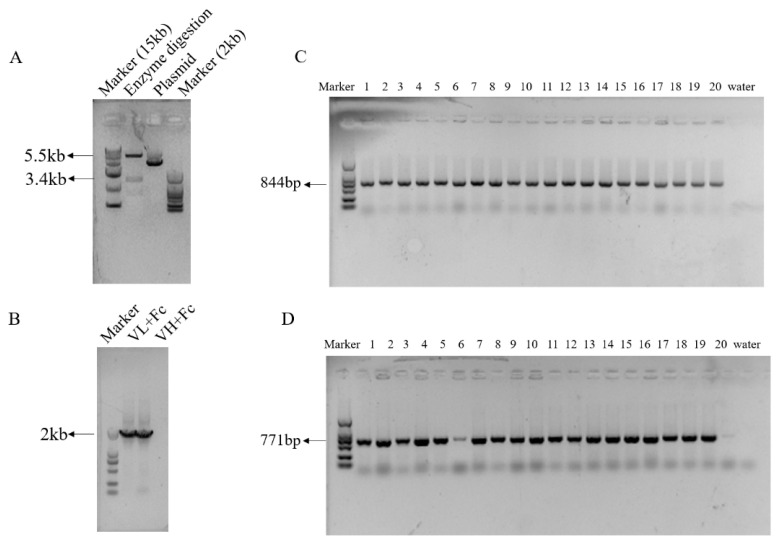
Construction of eukaryotic expression vectors containing PEDV neutralizing antibody genes. (**A**) pCI-Neo-hTERT digested with *Nhe* I and *Sal* I, and pCI-Neo-hTERT uncut. Empty vector is 5.5 kb. (**B**) *Nhe* I and *Sal* I digestion of pCI-anti-PEDV-VL and pCI-anti-PEDV-VH., VL-Fc and VH-Fc are 2 kb. (**C**,**D**) Colony PCR products from cells transfected with pCI-anti-PEDV-VL and pCI-anti-PEDV-VH. VL-Fc is 844 bp and VH-Fc is 771 bp.

The genes for the light chain (VL) and heavy chain (VH) of anti-PEDV mAb-2 were linked to a fragment crystallizable (Fc) by fusion PCR. *Nhe* I and *Sal* I restriction sites were inserted at the 5′ and 3′ ends respectively of the anti-PEDV VL and VH genes before ligating into pCI-Neo-hTERT vectors. Figure 5A shows an agarose gel with *Nhe* I and *Sal* I digested empty vector and undigested empty vector. Figure 5B shows a *Nhe* I and *Sal* I restriction digest of pCI-anti-PEDV-VL and pCI-anti-PEDV-VH. For each vector, twenty transfected colonies were selected for colony PCR. The amplification length of VL-Fc was 844 bp and VH-Fc was 771 bp. Figure 5C,D show the PCR results. Because the antibody gene has been patented, primer sequences and Sanger sequencing results are not shown.

### 3.4. Generation of HEK293 Cells Expressing Anti-PEDV mAb-2 Genes

**Figure 6:**
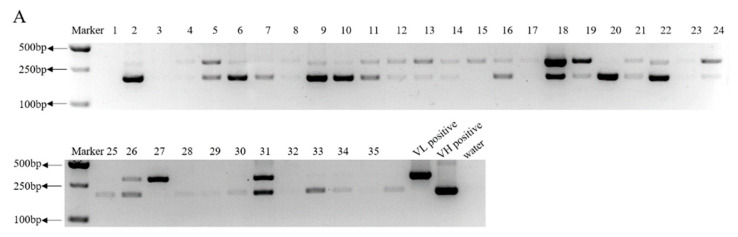

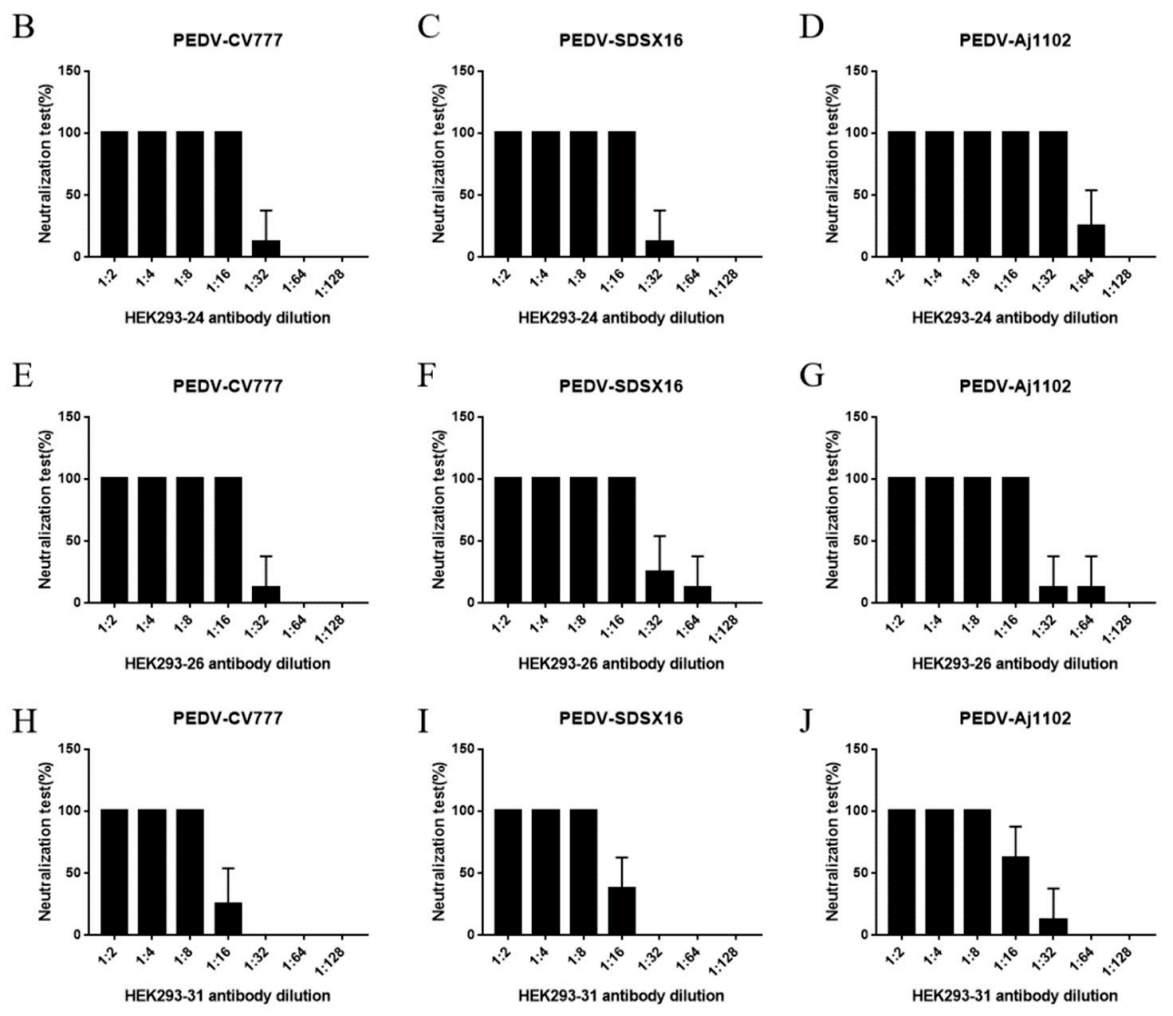
HEK293 cells expressing anti-PEDV mAb-2 genes. (**A**) PCR identification of HEK293 cells expressing VL-Fc at 548 bp and VH-Fc at 410 bp from clones 5, 18, 21, 22, 24, 26, and 31. pCI-anti-PEDV-VL mAb-4 and pCI-anti-PEDV-VH mAb-12 were positive controls. (**B**–**J**) Neutralization of PEDV-CV777, PEDV-SDSX16 and PEDV-Aj1102 by supernatants from clones 24, 26, and 31.

After RT-PCR identification, HEK293 cells expressing anti-PEDV mAb-2 genes were expanded and cell supernatants were collected for neutralization assays. Figure 6A shows of the 36 clones collected from the co-transfected HEK293 cells, 7 were positive for VL-Fc and VH-Fc. Supernatants from these 7 cultures, see lanes 5, 18, 21, 22, 24, 26, and 31 were tested for neutralization activity against PEDV-CV777, PEDV-SDSX16 and PEDV-Aj1102. As shown in Figure 6B–J, all strains were neutralized by supernatants from clones 24, 26, and 31 at a dilution of 1:32, 1:16, and 1:8 respectively. Supernatant from clones 5, 18, 21 and 22 had no neutralization activity, and they acted as negative controls (Table S3).

### 3.5. Oral Administration of Anti-PEDV mAb-2 Inhibits PEDV Infection in Piglets

**Figure 7:**
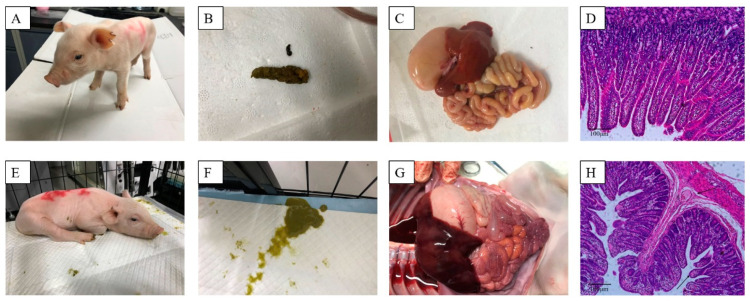
PEDV challenge. The feces, overall anatomical changes, and histopatholog of piglets. (**A**–**D**), group A piglets. (**E**–**H**), group B piglets. Black arrow indicates the intestinal villous epithelium and villous epithelial cells (**H**).

After 1–3 days of cohabitation, all group B piglets (those that received DMEM) and all the additional 12 piglets (group C and group D) began exhibiting mild disease symptoms, such as the loss of appetite and softening of feces. These symptoms worsened gradually over the course of the experiment. Between 7 and 10 days of cohabitation, the piglets had stopped eating, had severe watery diarrhea and were vomiting, the most severely affected piglets presented with hypothermia, weight loss, and death. Figure 7A–D shows the group A piglets; they exhibited a normal mental state, were energetic, and had dry solid feces. In short, group A piglets appeared healthy. Their small intestinal tissues and intestinal villous epithelia were normal. Figure 7E–H shows the group B piglets. They exhibited a depressed mental state, were lethargic, and had severe watery diarrhea. Their intestinal walls were thin and congested, intestinal cavitied were filled with yellow contents, and there were undigested milk clots were on the stomach wall. The intestinal villous epithelium was shed and the cytoplasmic vacuolization of villous epithelial cells was serious.

**Figure 8:**
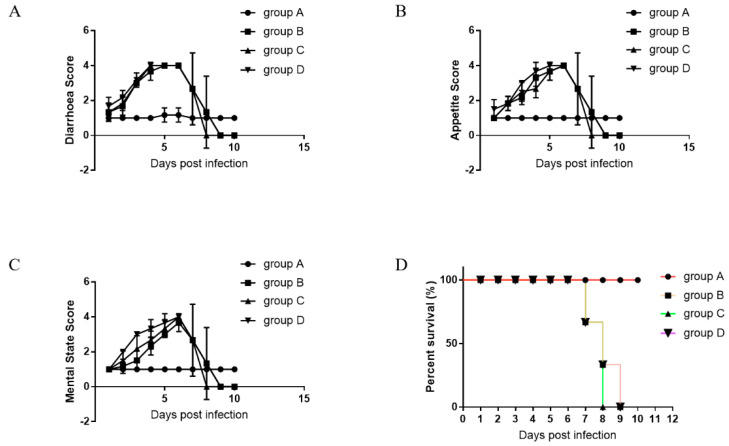
Oral administration of anti-PEDV mAb-2 inhibits infection in piglets. (**A**–**C**) Symptom scores for diarrhea, appetite and mental state respectively. (**D**) Percent survival of the tested piglets.

Symptom scores were used to assess the course of the disease. The group B piglets and those housed with them first showed symptoms after 1 day of cohabiting, while group A piglets showed no disease symptoms during the course of the experiment (Figure 8A–C). Detail symptomatic scores are shown in Table S4. All piglets in group A survived the 10 days of cohabitation, while the survival rates of piglets in group B and PED positive piglets (group C and group D) were 0%. The first piglet died at 7 days post-infection (dpi) (Figure 8D).

**Figure 9:**
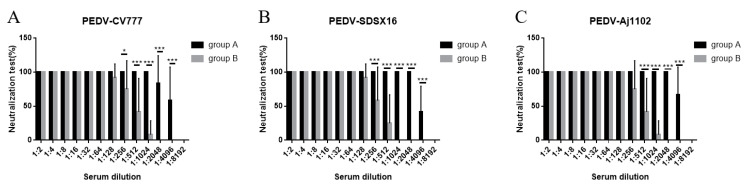
Neutralization of PEDV-CV777 (**A**), PEDV-SDSX16 (**B**), and PEDV-Aj1102 (**C**) by serum from piglets in groups A and B. Results are expressed as the mean values from triplicate wells. Data are shown as mean ± SEM (* *p* < 0.05, ** *p* < 0.01, *** *p* < 0.001).

Serum neutralizing antibody levels against PEDV-CV777, PEDV-SDSX16, and PEDV-Aj1102 was determined by neutralization assays. For 100% neutralization of PEDV-CV777, the maximum dilution from group A piglets was 1:1024, from group B piglets it was 1:64. For 100% neutralization of PEDV-SDSX16 and PEDV-Aj1102, the maximum dilution from group A piglets was 1:2048, from group B piglets was 1:64 and 1:128 respectively (Figure 9A–C).

The presence of PEDV was determined post mortem. vRNAs were isolated from small intestinal tissues, and the reverse transcripted cDNAs were used as PCR templates (Oligonucleotides used for PCR were in Table S5). At the time of their death, each piglet in group B was PEDV positive, upon euthanasia all piglets in group A were PEDV negative (Figure S1). The feces of piglets in both group A and group B were inoculated onto Vero cells after filtration and centrifugation. Vero cells inoculated group B feces were shrunken and shedding from the plate after 72 h, Vero cell inoculated with group A feces appeared normal and fully attached after the same time (Figure S2).

## 4. Discussion

PEDV is a global pathogen in pigs, its incidence rate is high in pigs of all ages and its mortality rate is nearly 100% in suckling piglets. PEDV has created serious economic losses to the swine industry [24]. Therefore, the development of an antibody that can stably exert antiviral function in piglets is an urgent need for pig industries worldwide.

Because of the high variability of PEDV, which is characterized by deletions, insertions, and amino acid substitutions in S gene, traditional vaccines provide only limited cross-reactivity [25]. The S protein is a type I glycoprotein that plays an important role in virus attachment, entry, receptor binding, cell membrane fusion, and induction of neutralizing antibodies, and these neutralizing epitopes have been identified for PEDV and TGEV [6,26]. The S protein is the main target of PEDV neutralizing antibodies, and various studies have shown that neutralizing antibodies can be produced by vaccines that stimulate the expression of S proteins [27,28]. Currently, Cruz et al., Sun et al., Li et al. and Okda et al. identified four neutralizing epitopes of the S protein from 1994 to 2017 [26,29,30,31,32]. Live viral vaccines and inactivated vaccines based on PEDV G1 strains protect only part of pigs from new variant strains [13]. Liu et al. have reported that PEDV G2 strain-based vaccines offer a promising addition to the fight against pandemic PEDV strains [33].

S-INDEL Iowa106 can neutralize the original American PEDV strain [34]. Piglets born from sows that were contact exposed to the S-INDEL variant PEDV can partially resist the challenge to traditional American PEDV strains [35]. S-INDEL induced partial protective immunity against the original US PEDV strain [36]. Studies have shown that, sows exposed to G1b S INDEL PEDV 7 months before delivery, then exposed to G2b non-S INDEL PEDV on day 109 of gestation can provide passive protection for piglets from G2b non-S INDEL PEDV for up to seven months, and the mortality rate was 0%, diarrhoea incidence rate decreased by 57%. The average mortality rate of piglets born in non-immune sows was 33%, the diarrhoea incidence was 100% [37].

In this study, we prepared an anti-PEDV monoclonal antibody (anti-PEDV mAb-2), and tested its neutralization activity against one PEDV G1 strain and two PEDV G2 strains. We found that there was no significant difference in neutralization titer among the three strains. At a dilution of 1:32, anti-PEDV mAb-2 effectively neutralized each strain. This result demonstrates that anti-PEDV mAb-2 has broad-spectrum activity and that our strategy can begin to address the limitations of conventional vaccines.

PEDV vaccines have been widely used in many large-scale pig farms, and the morbidity and mortality of vaccinated pigs are lower than on farms that do not vaccinate [13]. The poor immunity elicited by conventional PEDV vaccines, is their route of administration [2,38]. Newborn piglets are generally passively protected, obtaining antibodies from colostrum and sow’s milk, but proteases in the gastrointestinal tract of piglets degrade these antibodies, and thus absorbable IgA is reduced. Therefore, artificial passive immunity, by oral ingestion of antibodies, is an attractive way to confer increased resistance to PEDV [1,18,39]. In this study, newborn piglets orally dosed with an anti-PEDV mAb that we produced, successfully resisted the PEDV challenge. Because colostrum contains PEDV neutralizing antibodies [40], we used piglets that had not ingested colostrum. The results of the oral antibody-PEDV challenge experiment showed that our anti-PEDV mAb-2 has potential as a commercial vaccine; dosing is simple and it offers significant protection after one oral dose.

There have been several studies on the preparation of PEDV neutralizing antibodies [26,29,32], however, none have reported using an IgG antibody to orally dose piglets in order to prevent disease. Major hurdles to industrial antibody production have been high production costs and long production cycles. Here, we constructed HEK293 cell lines that secrete PEDV neutralizing antibodies comparable to those of hybridoma cells. This strategy makes it possible to produce larger amounts of neutralizing antibodies with lower production costs.

## 5. Conclusions

In conclusion, we demonstrated that anti-PEDV mAb-2 is efficiently expressed in mammalian cells and effectively prevents PEDV in orally dosed newborn piglets. To the best of our knowledge, this is the first study to demonstrate the preventive effect of feeding anti-PEDV antibodies to newborn piglets, indicating that PEDV neutralizing antibodies have considerable potential to slow, or stop, the spread of PEDV among pigs and alleviate the economic burden caused by the disease.

## Figures and Tables

**Table 1:** Scoring criteria for clinical symptoms in infected piglets.

Observation Projects	Evaluation Criteria	Score
A. Diarrhoea	Normal	1
	Fecal softening	2
	Soft stool with mild watery diarrhea	3
	Severe watery diarrhoea	4
B. Appetite	Normal	1
	Reduced appetite	2
	Poor appetite	3
	No appetite	4
C. Mental state	Normal	1
	Lethargic	2
	Often lying down and occasionally stand	3
	Barely breathing	4

## Data Availability

The data presented in this study are available on request from the corresponding author.

## Supplementary Materials

The following are available online at https://www.mdpi.com/1999-4915/13/3/472/s1, Figure S1: PCR identification of pathogens of piglets, Figure S2: Morphology of Vero cells after incubating with piglets feces., Table S1: Oligonucleotides used for PCR, Table S2: Genotype of heavy and light chains of PEDV mAbs, Table S3: Neutralization of PEDV-CV777, PEDV-SDSX16 and PEDV-Aj1102 by supernatants from clones 5, 18, 21, and 22. Table S4: The scores of diarrhoea, appetite and mental state, Table S5: Oligonucleotides used for PCR.
