# Supplementary material for: Expression and Purification of a PEDV-Neutralizing Antibody and Its Functional Verification
Source: Viruses. 2021 Mar 12;13(3):472. doi: 10.3390/v13030472 (PMC7999980; doi:10.3390/v13030472)
Supplement: Supplementary file 1 [file viruses-13-00472-s001.zip › Supplementary Table.docx]

**Table S1.** Oligonucleotides used for PCR.

| **Primer Name** | **Sequence 5’ -3’** | **Amplicon size (bp)** |
| --- | --- | --- |
| PEDV-F | AATTCATGACCATAGCATCG | 1052 |
| PEDV-R | TCGACCTAAATATTCCCAGTAACCG |  |
| TGEV-F | ACTAAGCTTTTAGCTTACATCACATGGCG | 2220 |
| TGEV-R | ACTGGATCCATGAAAAAATTATTTGTGG |  |
| PCV-F | CATCTTCAACACCCGCCTCT | 518 |
| PCV-R | GGATATTGTATTCCTGGTCGTAT |  |
| PRV-F | GCGTCGGGGTCCTCGTTCT | 1109 |
| PRV-R | CACGTGAACGACATGCTGA |  |

**Table S2.** Genotype of heavy and light chains of PEDV mAbs.

|  | **1** | **2** | **15** | **18** | **21** |
| --- | --- | --- | --- | --- | --- |
| Heavy Chain | G2a、G2b | G2a | G1 | G1 | G1 |
| Light Chain | κ | κ | λ | κ | κ |

**Table S3.** Neutralization of PEDV-CV777, PEDV-SDSX16 and PEDV-Aj1102 by supernatants from clones 5, 18, 21, and 22.

|  | **5** | **18** | **21** | **22** |
| --- | --- | --- | --- | --- |
| PEDV-CV777 | - | - | - | - |
| PEDV-SDSX16 | - | - | - | - |
| PEDV-Aj1102 | - | - | - | - |

^1^ There was no neutralization even with undiluted supernatants.

**Table S4.** The scores of diarrhoea, appetite and mental state.

| **DATE** | **Group A** | | | | | | **Group B** | | | | | | **Group C** | | | | | | **Group D** | | | | | |
| --- | --- | --- | --- | --- | --- | --- | --- | --- | --- | --- | --- | --- | --- | --- | --- | --- | --- | --- | --- | --- | --- | --- | --- | --- |
|  | **A1** | **A2** | **A3** | **A4** | **A5** | **A6** | **B1** | **B2** | **B3** | **B4** | **B5** | **B6** | **C1** | **C2** | **C3** | **C4** | **C5** | **C6** | **D1** | **D2** | **D3** | **D4** | **D5** | **D6** |
| 1 | 1,1,1 | 1,1,1 | 1,1,1 | 1,1,1 | 1,1,1 | 1,1,1 | 1,1,1 | 1,1,1 | 2,1,1 | 2,1,1 | 1,1,1 | 1,1,1 | 1,1,1 | 1,1,1 | 2,1,1 | 1,1,1 | 1,1,1 | 2,1,1 | 1,1,1 | 2,2,1 | 2,2,1 | 1,1,1 | 2,1,1 | 2,2,1 |
| 2 | 1,1,1 | 1,1,1 | 1,1,1 | 1,1,1 | 1,1,1 | 1,1,1 | 1,1,1 | 1,2,1 | 2,2,1 | 2,2,1 | 2,2,1 | 2,2,2 | 2,2,1 | 1,1,1 | 2,2,2 | 2,2,1 | 2,2,2 | 2,2,2 | 2,2,2 | 2,2,2 | 3,2,2 | 2,2,2 | 2,1,2 | 2,2,2 |
| 3 | 1,1,1 | 1,1,1 | 1,1,1 | 1,1,1 | 1,1,1 | 1,1,1 | 3,2,1 | 3,2,2 | 3,2,2 | 3,2,1 | 3,2,1 | 3,3,2 | 3,2,1 | 3,2,2 | 3,3,3 | 3,2,2 | 3,3,2 | 3,3,3 | 3,3,3 | 3,3,3 | 4,3,3 | 3,3,3 | 3,3,3 | 3,3,3 |
| 4 | 1,1,1 | 1,1,1 | 1,1,1 | 1,1,1 | 1,1,1 | 1,1,1 | 4,3,2 | 4,3,2 | 3,4,3 | 3,3,2 | 4,3,2 | 4,4,3 | 4,2,2 | 4,3,3 | 4,3,3 | 4,2,2 | 4,3,3, | 4,3,3 | 4,3,3 | 4,4,3 | 4,4,4 | 4,4,3 | 4,3,3 | 4,4,4 |
| 5 | 1,1,1 | 1,1,1 | 1,1,1 | 1,2,1 | 1,1,1 | 1,1,1 | 4,4,3 | 4,3,3 | 4,4,3 | 4,4,3 | 4,3,3 | 4,4,3 | 4,3,3 | 4,4,3 | 4,4,4 | 4,3,3 | 4,4,3 | 4,4,4 | 4,4,4 | 4,4,3 | 4,4,4 | 4,4,4 | 4,4,3 | 4,4,4 |
| 6 | 1,1,1 | 2,1,1 | 1,1,1 | 1,1,1 | 1,1,1 | 1,1,1 | 4,4,4 | 4,4,3 | 4,4,4 | 4,4,4 | 4,4,3 | 4,4,4 | 4,4,4 | 4,4,4 | 4,4,4 | 4,4,4 | 4,4,4 | 4,4,4 | 4,4,4 | 4,4,4 | 4,4,4 | 4,4,4 | 4,4,4 | 4,4,4 |
| 7 | 1,1,1 | 1,1,1 | 1,1,1 | 1,1,1 | 1,1,1 | 1,1,1 | 0,0,0 | 4,4,4 | 4,4,4 | 0,0,0 | 4,4,4 | 4,4,4 | 4,4,4 | 4,4,4 | 0,0,0 | 4,4,4 | 4,4,4 | 0,0,0 | 4,4,4 | 4,4,4 | 0,0,0 | 4,4,4 | 4,4,4 | 0,0,0 |
| 8 | 1,1,1 | 1,1,1 | 1,1,1 | 1,1,1 | 1,1,1 | 1,1,1 | 0,0,0 | 0,0,0 | 4,4,4 | 0,0,0 | 0,0,0 | 4,4,4 | 0,0,0 | 0,0,0 | 0,0,0 | 0,0,0 | 0,0,0 | 0,0,0 | 0,0,0 | 4,4,4 | 0,0,0 | 0,0,0 | 4,4,4 | 0,0,0 |
| 9 | 1,1,1 | 1,1,1 | 1,1,1 | 1,1,1 | 1,1,1 | 1,1,1 | 0,0,0 | 0,0,0 | 0,0,0 | 0,0,0 | 0,0,0 | 0,0,0 | 0,0,0 | 0,0,0 | 0,0,0 | 0,0,0 | 0,0,0 | 0,0,0 | 0,0,0 | 0,0,0 | 0,0,0 | 0,0,0 | 0,0,0 | 0,0,0 |
| 10 | 1,1,1 | 1,1,1 | 1,1,1 | 1,1,1 | 1,1,1 | 1,1,1 | 0,0,0 | 0,0,0 | 0,0,0 | 0,0,0 | 0,0,0 | 0,0,0 | 0,0,0 | 0,0,0 | 0,0,0 | 0,0,0 | 0,0,0 | 0,0,0 | 0,0,0 | 0,0,0 | 0,0,0 | 0,0,0 | 0,0,0 | 0,0,0 |

^1^ Numbers in each box of the table mean diarrhea score, appetite score and mental state score in turn.

**Table S5.** Oligonucleotides used for PCR.

| **Primer Name** | **Sequence 5’ -3’** | **Amplicon size (bp)** |
| --- | --- | --- |
| PEDV-F2 | TTGCAAGTGGCGCTGTGATT | 442 |
| PEDV-R2 | GACACCCTGGTTTTCACCAA |  |
