# Supplementary material for: Expression and Purification of a PEDV-Neutralizing Antibody and Its Functional Verification
Source: Viruses. 2021 Mar 12;13(3):472. doi: 10.3390/v13030472 (PMC7999980; doi:10.3390/v13030472)
Supplement: Supplementary file 1 [file viruses-13-00472-s001.zip › Supplementary Figure.docx]

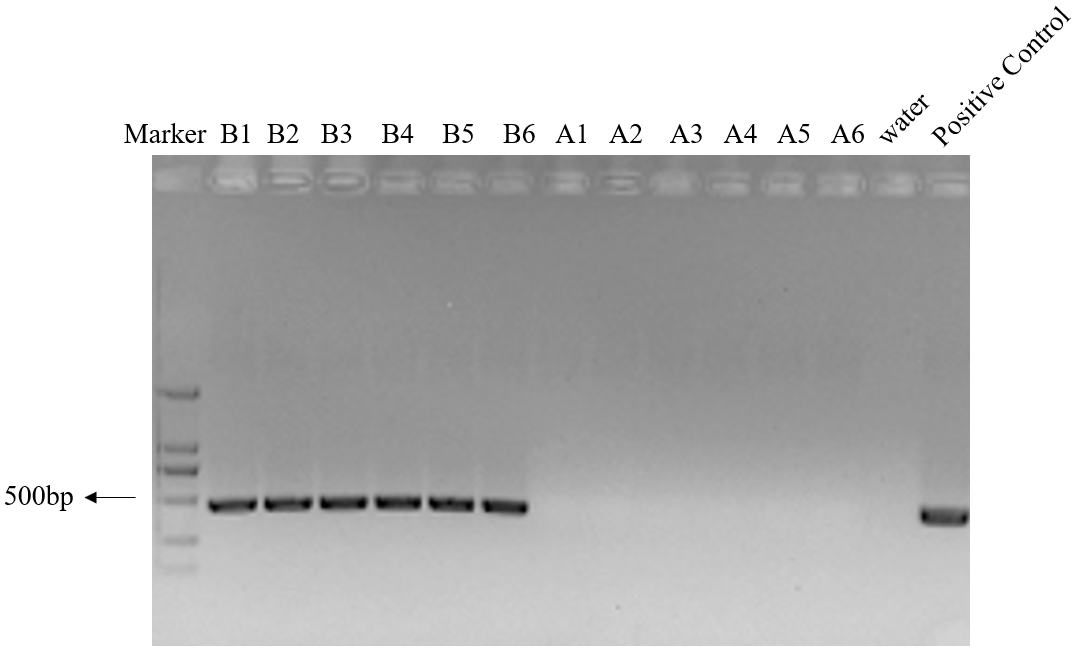


**Figure S1.** PCR identification of pathogens of piglets. A1-A6 represent 6 piglets in group A. B1-B6 represent 6 piglets in group B. SDSX16 is the positive control. The amplicon size was 442 bp.


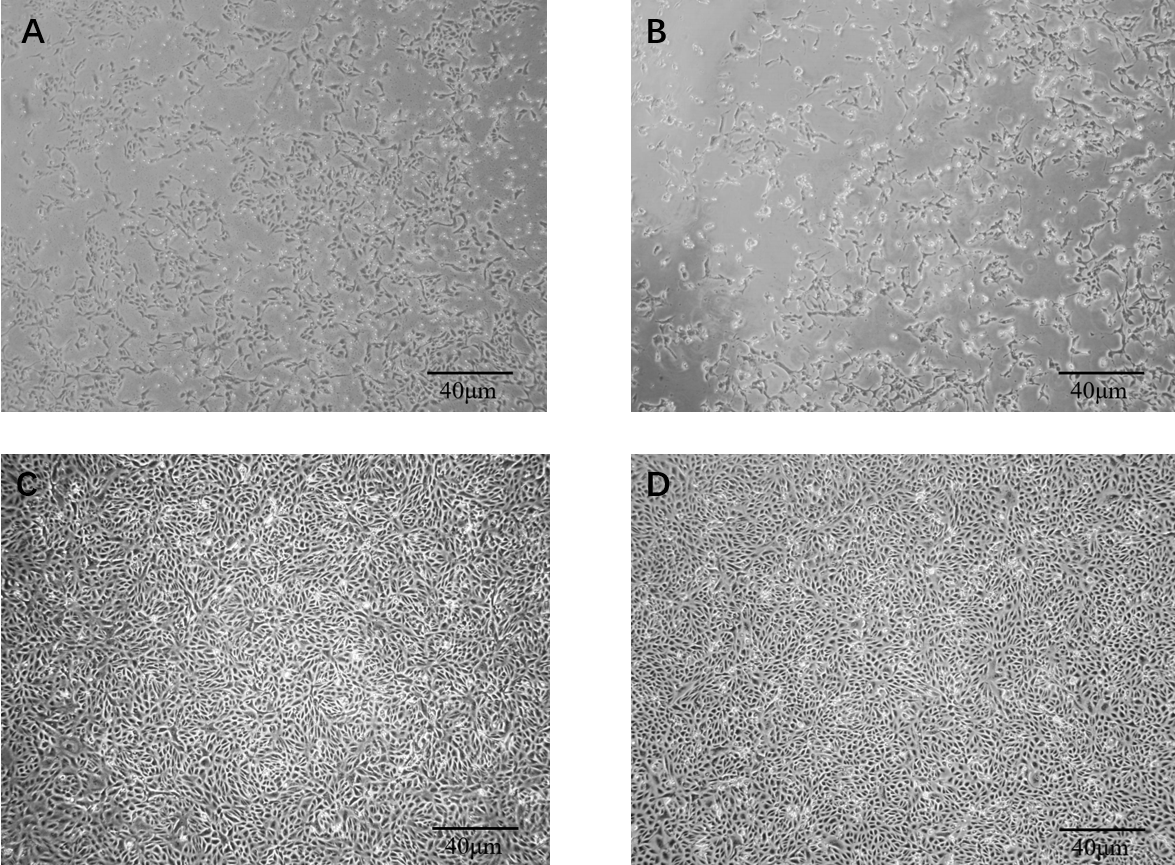


**Figure S2.** Morphology of Vero cells after incubating with piglets feces. Vero cells were infected with piglet feces from group A (C-D) and group B (A-B) after filtration and centrifugation. PEDV-specific CPE was monitored daily using an inverted microscope. A-B. Vero cells were shrunken and shedding from the plate after 72 hpi at P5 and P10 respectively. C-D. The morphology of Vero cells was normal after 72 hpi at P5 and P10 respectively.
